# System-Level Analysis of Alzheimer’s Disease Prioritizes Candidate Genes for Neurodegeneration

**DOI:** 10.3389/fgene.2021.625246

**Published:** 2021-04-06

**Authors:** Jeffrey L. Brabec, Montana Kay Lara, Anna L. Tyler, J. Matthew Mahoney

**Affiliations:** ^1^Department of Neurological Sciences, University of Vermont, Burlington, VT, United States; ^2^The Jackson Laboratory, Bar Harbor, ME, United States

**Keywords:** gene prioritization, machine learning, GWAS, Alzheimer’s disease (AD), network-based functional prediction, Alzheimer’s Disease Neuroimaging Initiative (ADNI)

## Abstract

Alzheimer’s disease (AD) is a debilitating neurodegenerative disorder. Since the advent of the genome-wide association study (GWAS) we have come to understand much about the genes involved in AD heritability and pathophysiology. Large case-control meta-GWAS studies have increased our ability to prioritize weaker effect alleles, while the recent development of *network-based functional prediction* has provided a mechanism by which we can use machine learning to reprioritize GWAS hits in the functional context of relevant brain tissues like the hippocampus and amygdala. In parallel with these developments, groups like the Alzheimer’s Disease Neuroimaging Initiative (ADNI) have compiled rich compendia of AD patient data including genotype and biomarker information, including derived volume measures for relevant structures like the hippocampus and the amygdala. In this study we wanted to identify genes involved in AD-related atrophy of these two structures, which are often critically impaired over the course of the disease. To do this we developed a combined score prioritization method which uses the cumulative distribution function of a gene’s functional and positional score, to prioritize top genes that not only segregate with disease status, but also with hippocampal and amygdalar atrophy. Our method identified a mix of genes that had previously been identified in AD GWAS including *APOE*, *TOMM40*, and *NECTIN2*(*PVRL2*) and several others that have not been identified in AD genetic studies, but play integral roles in AD-effected functional pathways including *IQSEC1*, *PFN1*, and *PAK2*. Our findings support the viability of our novel combined score as a method for prioritizing region- and even cell-specific AD risk genes.

## Introduction

The central goal of genome-wide association studies (GWAS) in Alzheimer’s disease (AD) is to identify novel candidate genes influencing risk for developing AD. Like other complex disorders, AD has highly polygenic risk, where hundreds or even thousands of small-effect alleles modify the probability of developing AD (Lee et al., 2013; Carmona et al., 2018). Fundamentally, this genetic complexity arises from the underlying biological complexity of AD, where all the major cell types of the brain and multiple highly differentiated brain structures have established roles in pathogenesis or symptom severity (Calderon-Garcidueñas and Duyckaerts, 2017; Jaroudi et al., 2017). To fully capture this biological complexity for genetic mapping, the international community has undertaken multiple strategies, including *case-control GWAS* and *imaging GWAS*, that capture distinct components of the genetic risk for AD. In particular, case-control GWAS is well powered to detect risk alleles but cannot ascribe these effects to specific brain pathologies. On the other hand, imaging GWAS can localize the effect of alleles, but these studies have limited sample size and, therefore, limited statistical power. In this study, we apply a *network-based gene reprioritization* (NGR) strategy that leverages mature functional prioritization methods to integrate AD risk-gene networks from case-control GWAS with imaging GWAS data to predict genes that specifically influence hippocampal and amygdalar atrophy.

The spectrum of AD risk alleles is well studied, particularly in European populations (Hu et al., 2017; Solomon et al., 2018; Jansen et al., 2019; Rajan et al., 2019; Andrews et al., 2020). Using gold-standard cognitive exams that provide robust *premortem* diagnoses of AD, modern case-control GWAS are powered to detect small-effect alleles using large cohorts. These efforts have culminated most recently in a meta-analysis of AD GWAS assessing the effect of 9,862,738 SNPs in 71,880 cases and 383,378 controls (Jansen et al., 2019). With such large-scale studies, it has been possible to detect 2,357 variants and 29 genes with genome-level significant associations to AD (Jansen et al., 2019). However, increasing population size has diminishing marginal returns. Newly resolved effects are ever weaker. Moreover, the functional role of these alleles cannot be localized to any of the relevant cellular or regional drivers of AD pathology based on case-control status alone. Nevertheless, with a valid AD diagnosis as an endpoint, the alleles mapped in case-control GWAS can be confidently attributed to AD risk.

As an alternative to large case-control studies, the Alzheimer’s Disease Neuroimaging Initiative (ADNI) uses structural magnetic resonance imaging (MRI) as a phenotype for GWAS (Wyman et al., 2013). In contrast to cognitive exams, which measure the complex emergent functions of distributed neural circuits, neuroimaging localizes particular structural pathologies. In principle, alleles that have a small overall effect on disease risk could have a comparatively stronger effect on critical pathologies, including hippocampal and amygdalar atrophy, that mediate the genetic risk factors for developing AD. However, MRI is expensive and time-consuming, so the ADNI sample size is limited to the thousands, not hundreds of thousands, of subjects. To date, 2272 patients have been recruited, a subset of 556 of which have both imaging and genotype data (ADNI-1 cohort) (Weiner et al., 2015). This dramatically limits statistical power relative to case-control GWAS. Moreover, while some longitudinal data have been gathered (Bhagwat et al., 2018), it is currently impossible to dissociate background developmental differences in brain structures from pathogenic changes due to AD. Thus, for example, alleles influencing the growth of the hippocampus cannot be distinguished from alleles that exacerbate hippocampal atrophy.

To leverage the independent strengths of case-control and imaging GWAS, we performed an integrative analysis. Using NGR with the well-powered case-control meta-GWAS (Jansen et al., 2019), we identified hippocampus- and amygdala-specific functional networks that were enriched for AD risk genes. We then used a novel approach to combine these functional results with imaging GWAS results for low hippocampal and amygdalar volume in patients with AD. By combining AD specificity from NGR with genetic influences on low hippocampal and amygdalar volume, we can prioritize high-confidence genes for AD-induced hippocampal and amygdalar atrophy.

The key insight to NGR is that the tail of low *p*-values from a GWAS is typically highly enriched for genes in disease-relevant biological processes, independent of whether most of those genes achieve genome-wide significance (Greene et al., 2015). For any choice of statistical cutoff there is a tradeoff between (*a priori* unknown) false positives and false negatives. In particular, genome-wide significance is a conservative threshold that has many false negatives. With a more liberal threshold, one captures more true positives at the cost of more false positives, with no way to discriminate one from the other using GWAS data alone. In order to distinguish likely true positives from false positives, NGR augments the GWAS statistical signals with functional gene-gene interactions. The essential idea of NGR is that true positive genes, by virtue of being functionally related to the disease, are likely to be functionally related to each other. By identifying subnetworks that are enriched for interactions among nominally significant GWAS genes, we can distinguish the likely true positives from spurious associations. Several approaches to NGR have been recently developed, including strategies based on support vector machines (SVM) (Greene et al., 2015), network diffusion (Li and Li, 2012), and Bayesian data integration (Wu et al., 2017). All methods return a *functional score* for every gene in the genome (a *reprioritization*) that measures how strongly each gene interacts with the nominally significant GWAS hits. Using NGR, many groups have shown significant improvements in disease gene prediction (Greene et al., 2015; Wu et al., 2017), including in AD (Song et al., 2016; Yao et al., 2017).

In this study, following Guan et al. (2010), we used an ensemble of SVMs to reprioritize AD risk genes from case-control GWAS using hippocampus- and amygdala-specific functional networks. We then integrated these tissue-specific functional scores with imaging GWAS *p*-values for hippocampal and amygdalar volume. Using a combined score based on the joint cumulative density function of functional scores and imaging GWAS *p*-values, we prioritized candidate genes for hippocampal and amygdalar atrophy in AD and defined the putative AD gene networks in which these candidate genes function.

## Materials and Methods

### Data

**FIGURE 1:**
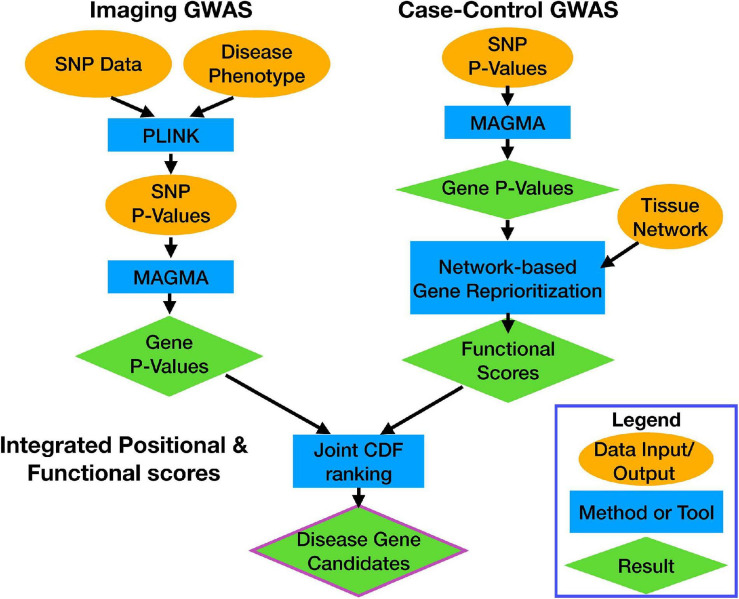
Workflow overview. Each data stream, and the calculation of the integrated score, are indicated by the bolded labels. Each section shows how data (orange ovals) were processed by computational tools (blue rectangles) to get results (green diamonds). Arrows indicate flow of information through the pipeline.

We used two distinct GWAS data sets and processed them through separate pipelines (Figure 1). The first data set is from the ADNI database and includes genotype and structural MRI imaging data^1^. The ADNI was launched in 2003 as a public–private partnership, led by Principal Investigator Michael W. Weiner, MD. The primary goal of ADNI has been to test whether serial MRI, positron emission tomography (PET), other biological markers, and clinical and neuropsychological assessment can be combined to measure the progression of mild cognitive impairment (MCI) and early AD. Identifying novel biomarkers of AD will help aid clinicians and researchers develop effective treatments and interventions.

Alzheimer’s Disease Neuroimaging Initiative is the result of efforts of many co-investigators from a broad range of academic institutions and private corporations, and subjects have been recruited from over 50 sites across the United States and Canada. The initial goal of ADNI was to recruit 800 subjects but ADNI has been followed by ADNI-GO and ADNI-2. To date these three protocols, in addition to the ongoing ADNI-3, have recruited over 2200 adults, ages 55–90, to participate in the research, consisting of control, non-AD (CN) older individuals, people with early or late MCI (EMCI or LMCI), and people with early AD. The follow up duration of each group is specified in the protocols for ADNI-1, ADNI-2, and ADNI-GO. Subjects originally recruited for ADNI-1 and ADNI-GO had the option to be followed in ADNI-2. Thousands of longitudinal imaging scans (Jack et al., 2008; Jagust et al., 2010), performance on neuropsychological and clinical assessments (Petersen et al., 2010) and biological samples (Shaw et al., 2009) were collected at baseline and at follow-up visits for all or a subset of participants. Genome-wide genotyping data (Saykin et al., 2010) are available on the full ADNI sample. For up to-date information, see www.adni-info.org.

Freesurfer version 5.1 was used to extract hippocampal volume and amygdalar volume measures from the 1.5 T baseline MRI scans of the ADNI-1 participants as described previously (Risacher et al., 2013). The measurements were retrieved from the ADNI data archive.

Genotype data of all participants from ADNI-1 were downloaded, quality controlled, and imputed to get full coverage beyond the initial 600,000 SNPs available on the Illumina 610Quad platform. Initial QC was performed using PLINK 1.9^2^ (Chang et al., 2015). Genotype data were processed as follows: (1) Samples missing more than 10% of their genotype calls were removed (one person removed), (2) SNPs with a minor allele frequency (MAF) greater than 0.05 were filtered for samples missing greater than 5% of the genotype calls and those with an MAF less than 0.05 were filtered for samples missing greater than 1% of genotype calls (48,026 variants removed), (3) duplicated samples were removed (14,238 variants removed), (4) samples that failed Hardy–Weinberg Equilibrium (HWE) (*p* < 10^–7^) were filtered out (434 variants removed). After QC, we performed genotype imputation using BEAGLE 5.1^3^ (Browning et al., 2018). Briefly, genotype data were split by chromosome and each chromosome was mapped onto the appropriate reference genome (hg37) and imputed to the CEU 1000 Genomes Project (1000 Genomes Project Consortium et al., 2015) reference panel. Imputed chromosomes were recombined using PLINK 1.9 and underwent an additional round of QC following the procedures listed above (433 variants removed for not meeting HWE). After imputation, 14,403,717 variants and 683 samples passed QC. Hippocampal and amygdalar volumes were used as the phenotypes in two separate GWAS analyses. A total of 556 individuals had both genotyping data and imaging phenotype data (*n* = 120 AD, *n* = 261 MCI, *n* = 175 CN). Genome scans were performed using PLINK 1.9 using a linear regression model with covariates for age, sex, education, and intracranial volume (ICV), following the GWAS protocol of a recent ADNI study using a related network-based gene reprioritization approach (Song et al., 2016).

SNP-level *p*-values were mapped to gene level *p*-values using MAGMA^4^ (de Leeuw et al., 2015). SNPs were annotated to genes using the hg37 genetic reference and a 10 kb annotation window on either side of the gene. The window size was chosen to match that used for gene mapping the AD meta-GWAS study (Jansen et al., 2019). Of the 14,403,717 SNPs contained within the ADNI genotype data, a total of 6,989,349 SNPs mapped to 18,385 genes. The HV GWAS yielded 338 nominally significant genes and three genes that reached a Bonferroni–Holm corrected, genome-wide significant *p*-value (Supplementary File 1). The AV GWAS yielded 276 nominally significant genes and 1 gene that reached a Bonferroni–Holm corrected genome-wide significant *p*-value (Supplementary File 2).

The second data set we analyzed was the AD meta-GWAS study conducted previously (Jansen et al., 2019). In that study, Jansen et al. (2019) performed a meta-analysis on case-control AD data from four major studies including the Alzheimer’s disease working group of the Psychiatric Genomics Consortium (PGC-ALZ), the International Genomics of Alzheimer’s Project (IGAP), the Alzheimer’s Disease Sequencing Project (ADSP), and UK Biobank (UKB). This analysis resulted in 71,880 AD cases and 383,378 non-AD controls and 9,862,738 SNPs passing quality control. SNP associations were calculated by regression as follows:

(1) Logistic regression was used to calculate SNP association with case control phenotypes from ADSP, PGC-ALZ, and IGAP.
(2) Linear regression was used to calculate associations for a continuous phenotype from UKB (calculated as the number of parents with AD).
(3) Associations were adjusted for sex as well as age. However, the ADSP study did not use age as a covariate as the study group was highly enriched for older patients and inclusion of age as a covariate in that study eliminated true AD associations (see Methods: Data Analysis in Jansen et al., 2019).
(4) The first four ancestry principal components (PCs) were also used to adjust statistical associations. A total of 20 were calculated and more were used if they showed a strong association with the phenotype.
(5) For UKB 12 PCs, age, sex, genotyping array, and testing center were all used as covariates.

SNP summary statistics were downloaded from the Center for Neurogenomics and Cognitive Research website: https://ctg.cncr.nl/software/summary_statistics. We used MAGMA to compute gene-level *p*-values as above. Of the 13,367,299 SNPs contained within the meta-GWAS summary statistics, 6,536,525 mapped to a total of 18,456 genes. At a nominal level of significant (*p* < 0.01) the meta-GWAS had 735 significant genes, while a Bonferroni–Holm corrected *p*-value yielded 28 genome-wide significant genes (Supplementary File 3).

### Network-Based Gene Repositioning

To functionally score every gene in the genome for relevance to AD, we performed NGR. NGR requires two inputs: a set of positive examples of *disease-associated genes*, and a *functional network* encoding gene-gene interactions (*cf.* Greene et al., 2015). From these data, NGR uses the network to propagate the “disease-associated” annotation to genes that are well connected to the disease-associated gene set. In this study, we used nominally significant AD-GWAS genes (*p* < 0.01) from the MAGMA analysis of the meta-GWAS as disease-associated genes. For functional networks, we used the hippocampus and amygdala tissue-specific functional networks freely available for download at HumanBase^5^ (‘hippocampus_top’ and ‘amygdala_top’) (Wong et al., 2018). Briefly, these networks were generated using a regularized Bayesian knowledge integration based on tissue ontology and a combination of gene expression datasets from the Gene Expression Omnibus (Barrett et al., 2013) representing 20,868 conditions (Greene et al., 2015). Each functional network is a weighted network, where each pair of genes (*g*_*i*_, *g*_*j*_) is linked with a weight, *W*_*g_i g_j*_, encoding the predicted probability that those genes functionally interact in that tissue. We define a *feature vector*, *f*_*g*_, for each gene, *g*, in the genome as the vector of weights connecting *g* to the *n* AD-GWAS genes, *p*_1_,…,*p*_*n*_ (i.e., positive examples),

$$f_{g}=\left[W_{g\,p_{1}},\ldots ,W_{g\,p_{n}}\right].$$

Using these feature vectors, we trained an ensemble of 100 (linear) SVM classifiers to distinguish between AD-GWAS genes and the rest of the genes in the genome. Formally, this problem is an instance of *positive-unlabeled (PU) learning* (PU), as we only have positive examples of AD-relevant genes (i.e., GWAS hits), but the status of all other genes is unknown. In the PU learning setting, we can treat all unlabeled examples as negatives for the sake of training the model, with the understanding that many unlabeled examples are likely AD-associated genes (Elkan and Noto, 2008). For each of the 100 SVMs, we trained using all positive examples and a random, balanced set of unlabeled examples as putative negatives. Each SVM was cross-validated to optimize its cost hyperparameter, C, over a grid, as described previously (Tyler et al., 2019). Each model *M*_*i*_ assigns each gene, *g*_*j*_, a model-based, real-valued prediction score *M*_*i*_(*g*_*j*_), where large positive scores correspond to high confidence that the gene is a positive example and negative scores correspond to low confidence. To normalize prediction scores across models prior to aggregation, we computed an *unlabeled-predicted-positive rate* (UPPR) for each model, *M*_*i*_, and gene, *g*_*j*_, as,

$${\text{UPPR}}_{\text{ij}}=\frac{\begin{array}{c} \#\,\left\{g\in \,U\,n\,l\,a\,b\,e\,l\,e\,d|M_{i}\,\left(g\right)>M_{i}\,\left(g_{j}\right)\right\} \end{array}}{\begin{array}{c} \#\left\{g\in Unlabeled\right\}|M_{i}\left(g\right)>M_{i}\left(g_{j}\right)\}\\ +\#\,\left\{g\in \,U\,n\,l\,a\,b\,e\,l\,e\,d|{\text{M}}_{\text{i}}\,\left(g_{j}\right)>M_{i}\,\left(g\right)\right\} \end{array}}$$

where ‘#’ denotes the cardinality of a finite set. The UPPR is the PU-learning equivalent of the false positive rate, where lower values indicate higher confidence that a gene is functionally associated with the AD GWAS genes. We averaged UPPR over all models and took the negative logarithm to obtain a final *functional score*, *FS*(*g*_*j*_)

$$F\,S\,\left(g_{j}\right)=-\log_{10}\left(\frac{1}{100}\,\sum_{i=1}^{100}U\,P\,P\,R_{i\,j}\right).$$

The functional score ranges from zero to infinity, with higher values indicating greater confidence. Models were trained using the *e1071* R package (Meyer et al., 2019).

### Integrating Functional and Positional Scores

To integrate functional scores for AD-specificity with imaging GWAS *p*-values, we computed a novel *combined score* based on the empirical joint cumulative density function (CDF) of the two scores. Specifically, every gene, g, had a functional score *FS(g)*, and a positional score *PS*(*g*) = –log_10_(*p*_*g*_), where *p*_*g*_ is the MAGMA *p*-value for *g* in the imaging GWAS. To quantify how highly ranked a gene, *g*_*j*_, is along both measures simultaneously, we used the value of the empirical joint CDF as a combined score, *CS(g_*j*_)*,

$$\text{CS}\,\left({\text{g}}_{\text{j}}\right)=\frac{\#\,\left\{\text{g}\in \text{Genome}|\mathrm{FS}\,\left(\text{g}\right)<\text{FS}\,\left({\text{g}}_{\text{j}}\right)\,\&\text{PS}\,\left(\text{g}\right)<\text{PS}\,\left({\text{g}}_{\text{j}}\right)\right\}}{\text{N}},$$

where N is the number of genes in the genome. Note that this is equivalent to the probabilistic definition using the empirical joint distribution of the two scores. Thus, the combined score represents the probability that a randomly chosen gene in the genome will score lower on both measures than *g*_*j*_.

### Functional Enrichment Analysis

To compare the functional enrichments of ADNI imaging genetics *p*-values versus the combined scores, we used the g:GOSt tool in the *gprofiler2* R package to identify significantly enriched Gene Ontology terms (Kolberg et al., 2020). Specifically, we ranked all genes by either *p*-value or combined score and tested the significance of all Gene Ontology Biological Process (GO:BP) terms (Ashburner et al., 2000; Carbon et al., 2018). We then summarized the enriched term lists into high-level annotations using the REVIGO online ontology analysis tool (Supek et al., 2011). Finally, we plotted high-level annotations as pie charts using *ggplot2* (Wickham, 2016).

### Modularity and Gene Enrichment Analysis of Functional Networks

To visualize and interpret the outputs of our SVM predictions, we plotted sub-networks of high-ranking genes and performed enrichment analyses of network modules. For both the hippocampal and amygdalar networks, we extracted the sub-networks of genes with functional scores greater than two (i.e., average UPPR < 0.01). We visualized these sub-networks using force-directed layout (Jacomy et al., 2014) in Gephi^6^ (Bastian et al., 2009). We identified modules in this sub-network using maximum modularity as implemented in Gephi (Blondel et al., 2008). The list of genes in each module was then sorted by functional score and input to g:GOSt (Raudvere et al., 2019), resulting in significantly enriched Gene Ontology (Ashburner et al., 2000; Carbon et al., 2018), KEGG (Kanehisa and Goto, 2000), and Reactome (Jassal et al., 2019) terms. Network modules were annotated by manually curating a set of representative functional terms, and the full output g:GOSt can be viewed in Supplementary Files 4, 5.

### Code Availability

To ensure rigor and reproducibility of our results, all analysis code used in this study is freely available at https://github.com/MahoneyLabGroup/AD_NBFP.

## Results

### Hippocampal Volume, Amygdalar Volume, and AD Diagnosis Captured Distinct Genetic Signals

**FIGURE 2:**
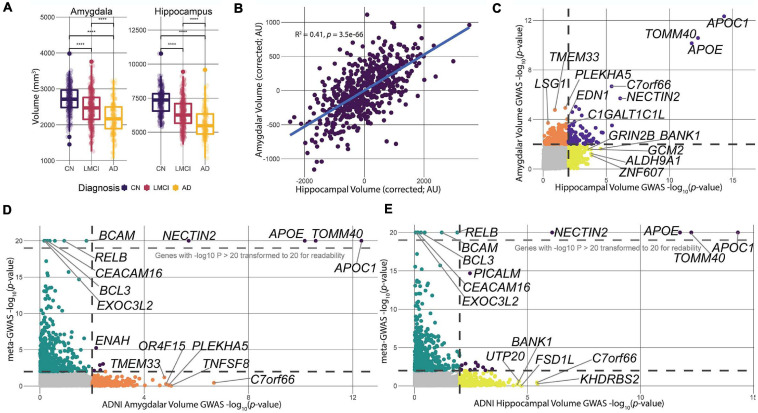
Data processing and comparison of hippocampal and amygdalar volume GWAS and meta-GWAS summary statistics. **(A)** We calculated the correlation between each of the three clinical diagnoses for ADNI-1 and the respective tissue volume measures. All three diagnoses showed significant (*p* < 2e-16) differences in average volume size across tissues. **(B)** Linear models predicting HV and AV were calculated to determine the correlation in size of the two structures. There was a significant, moderate correlation (*R*^2^ = 0.41, *p* = 3.6e-66) between the volumes of the two structures controlling for diagnosis at baseline, education, sex, ICV, and age. **(C)** Comparison of *p*-value distributions for the two GWAS volume measures. Dotted lines indicate a nominal significance cutoff of 0.01. **(D,E)** Comparison of *p*-value distributions between the meta-GWAS data and the respective volume GWAS data. Genes with log transformed *p*-values greater than 20 were transformed to 20. Black dotted lines indicate a nominal significance cutoff of 0.01.

The ADNI-1 dataset contains measures of hippocampal volume (HV) and amygdalar volume (AV) of patients and controls derived from structural MRI, as well as multiple relevant covariates: sex, age, educational attainment, and total ICV. Both of these brain volume measures correlated strongly with a patient’s clinical cognitive status (Figure 2A). Regional volumes were highest in control, non-AD (CN) subjects, lower in late mild cognitive impairment (LMCI) subjects, and lowest in patients with AD (Figure 2A). While there was overlap between the subgroups in HV and AV, the average size of each structure was significantly different between each clinical group (Figure 2A), as has been previously shown in prior ADNI work (Schuff et al., 2009; Whitwell et al., 2012).

The hippocampus and amygdala take part in overlapping limbic system neural pathways and are physically close to one another in the temporal lobe, suggesting that atrophy of each of these structures in AD could be highly correlated (Cavedo et al., 2011; Wang et al., 2016). To assess this, we corrected HV and AV for diagnosis at baseline, ICV, years of education, age, and sex using a linear model and computed the correlation of the residuals (Figure 2B). The residuals were significantly correlated (*R*^2^ = 0.41, *p* = 3.2e-66), indicating a significant, but moderate, correlation between the sizes of the two structures. The moderate correlation indicates that there are likely overlapping processes driving the size of these structures, but also biological processes that are unique to each. It is interesting to note that, after controlling for covariates, the distributions of HV and AV are unimodal and do not have any obvious subgroupings. Thus, for the remainder of the study, we treated HV and AV as quantitative traits.

To identify genetic drivers HV and AV in patients with AD, we used PLINK 1.9 (Chang et al., 2015) to statistically associate SNPs to HV and AV, and used MAGMA (de Leeuw et al., 2015) to integrate SNP-level association to gene-level associations (Figure 1). Overall, three genes—*APOC1, TOMM40*, and *APOE*—were significant after correcting for multiple comparisons for HV, and one gene—*APOC1*—was significant for AV. Furthermore, 338 and 276 genes were nominally significant at the *p* = 0.01 level for HV and AV, respectively. The top-ranked genes by *p*-value for both HV and AV were *APOC1*, *TOMM40*, and *APOE*, which all have well-established associations to AD (Zhou et al., 2014; Chiba-Falek et al., 2018; Zhao et al., 2018). Examining the nominally significant genes, we found that HV and AV independently associated with a unique subset of genes (Figure 2C). For example, the gene *GRIN2B*, which plays a role in brain development and is a candidate gene for temporal lobe epilepsy and autism spectrum disorder due to its effects on the hippocampus (Parrish et al., 2013; Varghese et al., 2017), was nominally significant for HV but not AV. Conversely, the gene *EDN1*, which is a candidate gene antagonist for multiple system atrophy (Gu et al., 2018), was nominally significant for AV but not HV. These results suggest that large-effect genes may have pleiotropic effects on HV and AV, but also that separate pathways may be driving atrophy in particular structures.

The virtue of endophenotypic measures such as HV and AV is they can potentially resolve biologically specific components of a disease that are otherwise too convoluted with other disease mechanisms when considering disease status alone. However, because the ADNI data are cross-sectional, it is not clear *a priori* whether genetic effects on HV or AV relate to genetic differences in brain developmental or to AD-induced atrophy. To assess the concordance between gene associations for HV and AV associations with AD risk *per se*, we compared gene-level *p*-values for HV and AV to corresponding *p*-values from the AD meta-GWAS study recently published (Jansen et al., 2019) (Figures 2D,E). The Jansen et al. (2019) study is the largest AD meta-GWAS to date, and provides the most robust data set to identify any HV- or AV-specific hits influencing AD risk. Like the comparison between HV and AV *p*-values, the meta-GWAS shares several genome-wide significant genes with HV and AV (Figures 2D,E). Furthermore, the meta-GWAS shares some nominally significant genes with imaging GWAS, for example, *ENAH* with AV and *PICALM* for HV (Figures 2D,E). These overlapping hits, at a nominal significance level, suggest that at least some of the variation in HV and AV is potentially driven by factors influencing genetic AD risk.

### NGR Identified Distinct Hippocampal and Amygdalar Functional Gene Networks Connecting AD Risk Genes

As major components of AD pathology, genetic risk factors for AD-induced hippocampal and amygdalar atrophy are expected to be a subset of all AD risk factors. However, differences in sample size (i.e., statistical power) and study population between the case-control and imaging GWAS limit our ability to detect these overlapping associations. Nevertheless, we expect that, beyond specific shared gene associations between HV and AV and disease risk, risk genes for imaging endophenotypes should lie in AD risk gene pathways. To identify the hippocampal and amygdalar pathways involved in AD pathogenesis, we performed NGR using hippocampus- and amygdala-specific functional genomic networks (Wong et al., 2018) to rank every gene in the genome by how well they connect to AD-GWAS genes. Briefly, we trained an ensemble of SVM classifiers to distinguish between AD-GWAS genes and the rest of the genome using connection weights to AD-GWAS genes in the tissue networks as features (see section “Materials and Methods”). The output of this analysis was a ranked list of genes with each gene receiving a *functional score* (formally, the negative logarithm of the unlabeled-predicted-positive rate) that quantifies how well connected a gene is to AD-GWAS genes. As positive examples we used all genes that reached a nominal level of significance (*p* = 0.01) in the meta-GWAS dataset (*n* = 735 genes). The remaining genes were treated as unlabeled.

**FIGURE 3:**
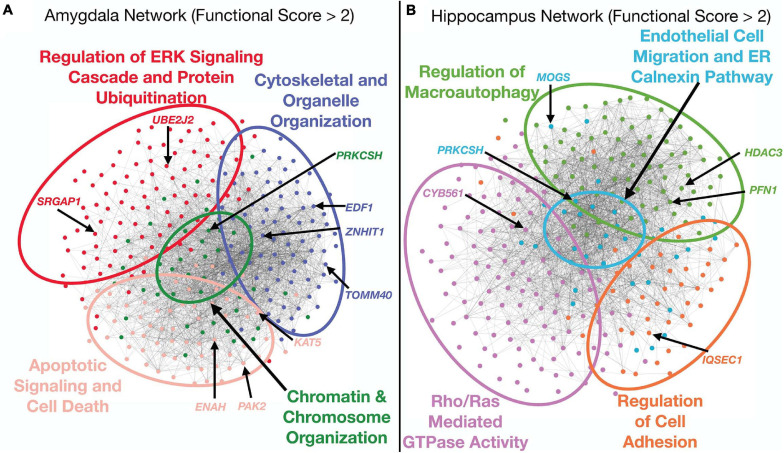
Analysis of top functional subnetworks. The functional subnetwork of genes with a functional scores greater than 2 were extracted from both tissue networks, run through a modularity algorithm, and the modules were functionally annotated using functional enrichment analysis by g:OSt. Several of the top hit genes from the combined ranking appeared in a diverse array of functional classifications. Each gene is colored by the functional module in which it is a member. Network edges were filtered to only include weights greater than 0.25 for visual clarity. **(A)** Amygdala sub-network analysis. The top functional sub-network for this tissue was enriched for genes in pathways that regulate apoptosis and cell death, cytoskeletal and organelle organization and chromosomal organization. **(B)** Hippocampus sub-network analysis. This top functional sub-network was enriched for genes involved in immune signaling as well as cell adhesion and ER regulation.

To aid the interpretation of top functional hits, we visualized the sub-networks of genes that had functional scores greater than 2 for the hippocampus and amygdala networks. We performed modularity analysis in Gephi (Bastian et al., 2009) and identified four modules in both sub-networks (Figure 3). We assigned functional annotations to the genes from each network module using g:GOSt (Raudvere et al., 2019). While the number of modules were the same for both tissue sub-networks, the functional annotations underscored distinct pathways. The hippocampus sub-network modules were enriched for genes taking part in *endothelial cell migration* (GO:0043542), *regulation of cell adhesion* (GO:0030155), *Rho/RAS mediated GTPase activity* (GO:0007266, GO:0046578), and *regulation of macroautophagy* (GO:0016241) (Figure 3A). The amygdala sub-network modules were enriched for genes involved in *regulation of the ERK signaling cascade and protein ubiquitination* (GO:0070372, GO:0030433), *cytoskeletal and organelle organization* (GO:0051493, GO:0033043), *chromatin and chromosome organization* (GO:0006325), and *apoptotic signaling and cell death* (GO:2001233, GO:0010941) (Figure 3B). These enrichments covered a diverse range of processes, some of which overlapped between tissues (e.g., *regulation of macroautophagy* and *apoptotic signaling and cell death*), while others appeared to be tissue-specific (e.g., *endothelial cell migration* in the hippocampus).

### Integration of Functional Scores With Imaging GWAS *p*-Values Predicted Risk Genes for AD-Induced Hippocampal and Amygdalar Atrophy

**FIGURE 4:**
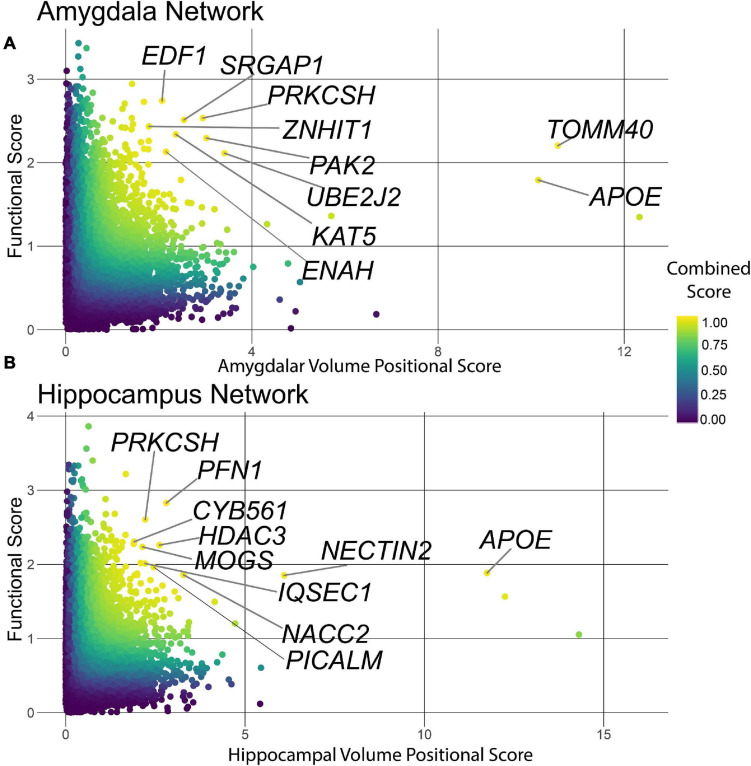
Combined score ranking. Points are colored on a gradient by combined score with yellow points scoring highest, and blue points scoring lowest. **(A)** The combined score plot for the amygdala tissue. Several of the top ranked genes were involved in the regulation gene transcription (*EDF1*) or the maintenance of organelles (*PRKCSH, UBE2J2*) and integrity of the synapse (*PAK2, ENAH*). **(B)** The combined score plot for the hippocampus tissue. Several genes were involved in processes required for the maintenance of the synapse (*PFN1, IQSEC1*) regulation of gene transcription (*HDAC3*) and proper ER regulation (*PRKCSH, MOGS*).

The HV and AV measurements are cross-sectional and cannot resolve whether a genetic association is due to AD-driven atrophy or a genetically encoded difference in brain development. Thus, the genes that associate with HV and AV need not necessarily associate with disease status. In order to identify genes that were simultaneously associated with HV or AV and functionally connected to AD disease risk, we computed a combined score using the joint cumulative density function of the imaging GWAS *p*-values and the functional scores from NGR. The resulting scores ranged continuously from zero to one, with values closer to one indicating a higher rank on both genetic and functional metrics. Plotting the functional score *vs*. the negative logarithm of the imaging GWAS *p*-value with a color gradient indicating each gene’s combined score, we see that some genes in the upper-right quadrant of the point cloud scored better than 95% of the genes in the genome on both axes (Figures 4A,B).

**FIGURE 5:**
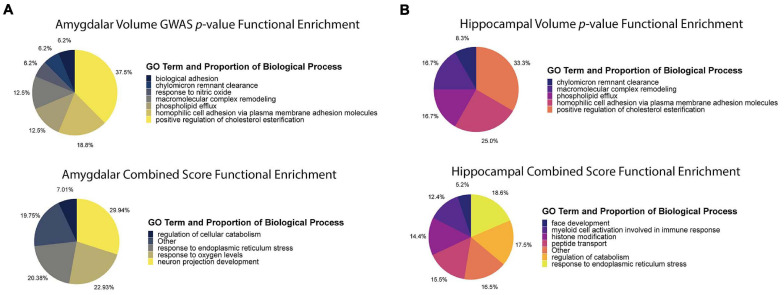
Functional enrichment analysis using *p*-value and combined score. **(A)** Functional enrichment analysis of amygdala volume GWAS *p*-value ranking and combined score rankings. The *p*-value functional enrichment analysis revealed terms like biological cell adhesion and macromolecular complex remodeling. By comparison, the functional enrichment analysis for the combined score revealed terms relating to ER stress, neuron projection development, and response to oxygen levels which are all pathways affected by AD pathophysiology. **(B)** Functional enrichment analysis of hippocampal volume GWAS *p*-value ranking and combined score rankings. The *p*-value functional enrichment analysis saw similar enrichments as the amygdala *p*-value functional enrichment analysis. The combined score functional enrichment analysis on the other hand, saw enrichment of pathways involved in development, ER stress, and immune response regulation.

The purpose of the combined score was to prioritize AD-specific genes and distinguish them from genes influencing HV and AV through developmental pathways. To establish a specific enrichment for AD-relevant pathways, we compared functional enrichments between ranking genes by *p*-value (ascending) and by combined score (descending). To summarize the large lists of enriched terms, we used REVIGO to compress the enrichments into representative high-level terms (Supek et al., 2011). For the hippocampus and amygdala (Figure 5), the *p*-value analyses revealed an enrichment for genes involved in cholesterol metabolism and cell adhesion. On the other hand, the combined score in the hippocampus was enriched for terms involved in the regulation of the immune response and cellular stress related to the endoplasmic reticulum (ER). Similarly, for the amygdala, the combined score was enriched for pathways involved in ER stress and neuron growth. These results demonstrate that the combined score prioritizes genes involved in AD-relevant functional pathways, distinct from those regulated by *APOE* (e.g., cholesterol metabolism) (Schliebs and Arendt, 2011; Heneka et al., 2015; Gerakis and Hetz, 2018).

Notably, while the combined score ranked genes involved in AD-relevant pathways highly, many of the top-10 genes have not been previously annotated to the disease (Tables 1, 2). High-scoring hippocampal genes are involved in actin regulation (*PFN1*, *IQSEC1*, *PAK2*), protein regulation in the ER (*MOGS* and *PRKCSH*), and transcriptional regulation (*HDAC3*). Highly ranked genes in the amygdala are involved in a wide range of processes, including regulation of proteins in the ER (*PRKCSH* and *UBE2J2*), transcription modification or cell cycle modulation (*KAT5*, *EDF1*, and *ZNHIT1*), and the maintenance and development of healthy synapses (*SRGAP1* and *PAK2*). The top 10 genes in both the hippocampus and amygdala were distributed throughout the NGR functional networks and were present in all functional modules (Figure 3).

**TABLE 1:** Brief descriptions of the top genes according to the combined score for the hippocampus.

Gene	Functional Score	*p*-value	Role (with PMID)
*PFN1*	0.00148	1.56E-03	Increased actin depolymerization in hippocampus of APP/PS1 mice indicates impaired synaptic plasticity (PMID: 31472195). Actin remodeling mediated by SGK1, a gene involved in spatial memory formation and consolidation (PMID: 31981651). Critical for proper PNS myelination, organization, and development (PMID: 24598164).
*HDAC3*	0.00549	2.44E-03	Nuclear HDAC3 is significantly increased in the hippocampus of 6- and 9-month-old APP/PS1 mice compared with age-matched wild-type C57BL/6 mice. Inhibition of HDAC3 in the hippocampus attenuated spatial memory deficits, and decreased amyloid plaque load and ABeta levels. Dendiritic spine density increased while microglial activation alleviated after HDAC3 inhibition. Over expression led to an increase in hipppocampal feels of Abeta, activation of microglia, and decreased dendritic spine density (PMID: 28771976).
*PRKCSH*	0.00249	6.06E-03	Colocalizes with IP3Rs which mediate calcium release from the ER, specifically in hippocampal neurons. Additionally, *PRKCSH* enhances IP3-induced calcium release and has been found to regulate ATP-induced CA2(+ (PMID: 18990696).
***APOE*** (29107063)	0.0130	1.78E-12	Lipid transporter that binds to cell-surface receptors to aid in cholesterol transport and membrane homeostasis. It is present in a broad range of functional pathways within the CNS including synaptic plasticity, mitochondrial function, and neuroinflammation. Its epsilon 4 allele is one of the biggest risk factors for AD (PMID: 28434655).
*MOGS*	0.00581	7.21E-03	Located in the lumen of the ER where it performs N-linked glycosylation. Several mutations within the gene can lead to congenital diseases of glycosylation which can lead to major structural malformations within the brain, liver, lungs, and many other higher-order tissues and organs (PMID: 30587846).
***NECTIN2*** (29107063)	0.0141	8.12E-07	Also known as *PVRL2*, this gene is a component protein of adherens junctions between cells. Has wide ranging roles in cell signaling to natural killer cells to leukocyte transport in endothelial cells (PMID: 28062492).
***PICALM*** (19734902)	0.0109	3.52E-03	Involved in clathrin assembly. Two SNPs 5′ to the gene are associated with Reduced LOAD Risk (PMID: 19734902; 24162737; 19734903), but their functions have not yet been determined. It colocalizes with APP and over-expression of PICALM *in vivo* increases plaque deposition in AD transgenic mice (PMID: 22539346). Binds to autophagosomes, suggesting a role in autophagy mediated Abeta clearance (PMID: 24067654).
*NACC2*	0.0139	5.19E-04	Transcription repressor within the p53 pathway: inhibits the expression of MDM2 which stabilizes the expression of p53 an important tumor suppressor (PMID: 22926524).
*IQSEC1*	0.00974	6.14E-03	Loss of function affects a wide variety of actin-dependent cellular processes, including AMPA and NMDA receptor trafficking at synapses (PMID: 20547133). Mutations have led to intellectual disability and developmental delays in those affected (PMID: 31607425).
*CYB561*	0.00496	1.24E-02	An electron transporter critical for the conversion of dopamine to epinephrine and norepinephrine. A mutation in this gene, which disrupts the final production of norepinephrine, has been observed in families with severe orthostatic hypotension (PMID: 29343526).

*Genes in bold have been previously found in AD GWAS. PMIDs from supporting papers are included in parentheses next to bolded gene names.*

**TABLE 2:** Brief descriptions of the top genes according to the combined score for the amygdala.

Gene	Functional Score	*p*-value	Role (with PMID)
*PRKCSH*	0.0293	1.13E-03	Colocalizes with IP3Rs which mediate calcium release from the ER, specifically in hippocampal neurons. Additionally, *PRKCSH* enhances IP3-induced calcium release and has been found to regulate ATP-induced CA2+ (PMID: 18990696).
***TOMM40*** (29107063)	0.00626	2.66E-11	Mitochondrial membrane protein critical for transport of protein precursors into the mitochondria and is associated with mitochondrial dysfunction in AD. Further, it has recently been found to be associated with functional connectivity of brain regions via fMRI (PMID: 31568198). It is in LD with APOE.
*PAK2*	0.00508	9.50E-04	Haploinsufficiency of PAK2 has been observed to decrease synapse density, impair LTP, and drive autism related behaviors in mice (PMID: 30134165). Strong regulator of cellular senescence and organismal aging through gene-expression and the H3.3 nucleosome assembly (PMID: 31209047).
*SRGAP1*	0.00308	2.89E-03	A GTPase activator that works with CDC42 to negatively regulate neuronal migration. Interacts with ROBO1 to inactivate CDC42 (PMID: 11672528).
*UBE2J2*	0.00771	2.88E-04	Ubiquitination by this protein is a potential mechanism for endoplasmic reticulum-associated depredation (ERAD) (PMID: 19951915; 25083800).
*KAT5*	0.00459	4.31E-03	A histone acetyl transferase (HAT) that plays a role in DNA repair and apoptosis as well as signal transduction. Complexes with the intracellular domain of the cleaved APP products to form nuclear spheres which seem to have a role in cell-cycle regulation, but are not well understood (PMID: 27644079).
*EDF1*	0.00181	8.50E-03	Transcriptional regulator of PPAR-gamma which has a wide array of roles in combatting AD pathophysiology including amyloid clearance and metabolic regulation (PMID: 22109891, 24838579).
*ENAH*	0.00740	6.99E-03	Complexes with FE65 and that association may have an effect on APP biogenesis (PMID: 9407065). Also involved in actin polymerization and cell motility (PMID: 10069337, 10892743).
*ZNHIT1*	0.00368	1.63E-02	Induces arrest of cell cycle at G1 and CDK6 was strongly down-regulated by Znhit1 through transcriptional repression (PMID: 19501046). CDK6 is unregulated in patients in AD compared to non-AD controls (PMID: 26766955).
***APOE*** (29107063)	0.0162	7.00E-11	Lipid transporter that binds to cell-surface receptors to aid in cholesterol transport and membrane homeostasis. It is present in a broad range of functional pathways within the CNS including synaptic plasticity, mitochondrial function, and neuroinflammation. Its epsilon 4 allele is one of the biggest risk factors for AD (PMID: 28434655).

*Genes in bold have been previously found in AD GWAS. PMIDs from supporting papers are included in parentheses next to bolded gene names.*

## Discussion

**FIGURE 6:**
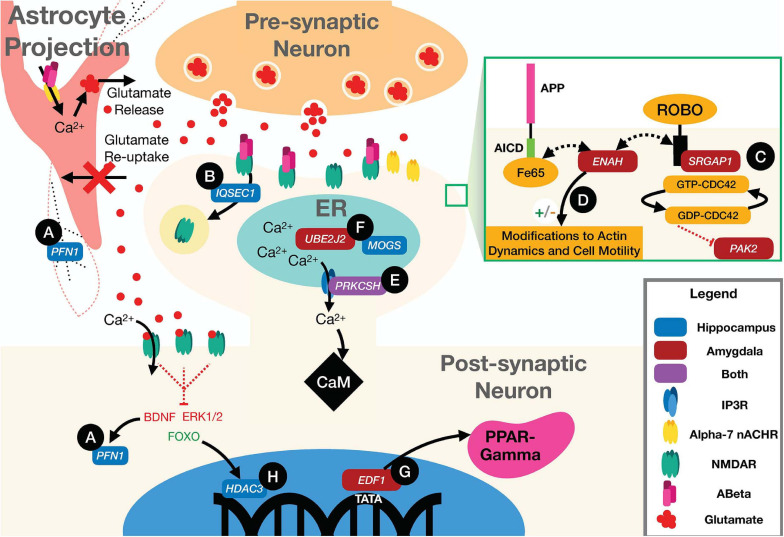
Illustration of neuronal and astrocytic pathways implicated in the disease according to the top scoring genes according to our integrated ranking. **(A)** Loss of signal from BDNF to *PFN1* downregulates the activity of the gene, impairing F-actin structure in the neuron and astrocyte. **(B)** *IQSEC1* mediates AMPAR and NMDAR receptor internalization. **(C)** *PAK2* activity is partially regulated by the CDC42 GTPase which is activated by binding of the SLIT protein to ROBO at the membrane. This activity can be interrupted by the activity of *SRGAP1* which inhibits ROBO signaling. **(D)** *ENAH* can bind to both ROBO and Fe65. When bound to ROBO it acts to inhibit actin polymerization and motility, but while bound to Fe65 and the AICD the ROBO pathway functions normally, promoting cell motility, and actin polymerization. **(E)** *PRKCSH* co-localizes with IP3Rs increasing calcium current through the channels, increasing cytosolic levels of Ca^2+^. **(F)** *UBE2J2* and *MOGS* are both involved in the proper regulation of intra-ER processes. Impairment of the proper activity of post-translational modification by *MOGS* could drive ERAD mediated by ubiquitination by *UBE2J2*. **(G)** *EDF1* is also a transcriptional regulator for PPAR-gamma. Activation of PPAR-gamma helps to regulate disturbed metabolic states and A plaque clearance. **(H)** *HDAC3* regulates transcription of many genes that have an effect on Aβ burden, microglial activation, and dendritic spine density.

As a complex disease, the genetic risk for AD is distributed over a wide variety of cellular and molecular pathways. Thus, the genetic architecture of AD is expected to be dominated by thousands of small-effect variants that each slightly perturb brain physiology toward a more AD-susceptible state, rather than a small set of highly penetrant mutations. Indeed, even the well-studied *APOE-E4* risk allele has an odds ratio of only 11.8 in the Caucasian population, which is by no means a certainty for any carrier (Jia et al., 2020). The value of genetic network analysis to the study of the architecture of complex disease, therefore, is to aggregate these many small perturbations into a pathway- and process-level description of the full disease. To this end, our results clearly implicate common mutations in many genes as perturbations of pathways that react to the aberrant accumulation of Aβ in the brain (Figure 6; discussed below). Far from being statistical noise, genes with nominally significant *p*-value from the imaging GWAS are enriched for AD-specific biology. Interestingly, the gene-level *p*-values largely did not replicate between imaging GWAS and the case-control meta-GWAS. It was only after identifying the relevant tissue-specific functional sub-networks with NGR that we could resolve the likely AD-specific genes for HV and AV. Validating any of these high-ranking genes as specifically influencing hippocampal or amygdalar atrophy is beyond the scope of this study, but many top hits have strong connections to well-established AD biology.

The pathognomonic signature of AD is the aggregation of amyloid β (Aβ) peptide into amyloid plaques in the brain. Beyond aggregating into plaques, however, Aβ is associated with a number of pathological processes, including loss of synaptic integrity (Rönicke et al., 2011; Parsons and Raymond, 2014; Wang and Reddy, 2016; Singh et al., 2017; Kang and Woo, 2019; Schaeverbeke et al., 2019) and dysregulating neuronal and astrocytic calcium channels (Yu et al., 2005; Rönicke et al., 2011; Parsons and Raymond, 2014; Lim et al., 2016; Wang and Reddy, 2016; Verkhratsky et al., 2017; Liu et al., 2019). At the astrocyte, Aβ has been shown to bind Alpha-7 nicotinic acetylcholine receptors (α7 nAChRs), causing an influx of calcium to the astrocyte and glutamate release into the synapse (Pirttimaki et al., 2013). At the synapse, Aβ has been shown to bind to *N*-methyl-D-aspartate receptors (NMDARs) preventing glutamate from activating the channel to allow an influx of calcium ions (Liu et al., 2019). Loss of current through NMDARs drives depression of synaptic strength at that synapse, as lower levels of calcium initially drive the endocytosis of α-amino-3-hydroxy-5-methyl-4-isoxasolepropionic acid receptors (AMPARs) and later NMDARs in the postsynaptic neuron (Tigaret et al., 2006; Yu et al., 2010). Loss of synaptic efficacy is a critical signal for synaptic pruning (Lüscher and Malenka, 2012), and an accumulated loss of synapses is one possible mechanism for loss of network function. Beyond synaptic pruning, Aβ is associated with a loss of synaptic integrity, where the neurotransmitters, such as glutamate, can leak out of the synapse and activate extra-synaptic receptors (Hardingham and Bading, 2010; Parsons and Raymond, 2014). It has been hypothesized that the high level of glutamate release by astrocytes leads to an increase in extra-synaptic glutamate signaling and excitotoxicity (Sattler et al., 2000; Hardingham and Bading, 2010; Parsons and Raymond, 2014; Wang and Reddy, 2016), which is hypothesized to both induce ER stress (Sokka et al., 2007; Concannon et al., 2008) and activate pro-apoptotic pathways (Hardingham et al., 2002), while antagonizing pro-survival pathways, particularly brain-derived neurotrophic factor (BDNF) signaling, leading to neuron death (Hardingham et al., 2002; Hardingham and Bading, 2010; Parsons and Raymond, 2014; Wang and Reddy, 2016). Thus, the accumulation of Aβ acts through multiple complex pathways—at the synapse, at the ER, and through transcriptional regulation—to cause atrophy of neural tissue. Importantly, our top-ranking genes in both the hippocampus and the amygdala act in these Aβ-response pathways.

### Multiple High-Ranking Genes Influence Synaptic Structure Through the Cytoskeleton

Altered synaptic structure and function are well-established in AD (Spires-Jones and Knafo, 2012; Pozueta et al., 2013; Chabrier et al., 2014; Price et al., 2014; Mango et al., 2019; Koller and Chakrabarty, 2020). The highest-ranking hippocampal gene, *PFN1* (Figure 6A and Table 1), encodes an actin-monomer binding protein that is known to regulate the cytoskeleton of neurites (Murk et al., 2012), but has also been shown to support the highly mobile *F*-actin in astrocytic projections that surround synaptic clefts (Schweinhuber et al., 2015). It has been associated with impaired synaptic plasticity and spatial memory in the *APP*/*PS1* mouse model of AD (Sun et al., 2019; Lian et al., 2020). Alterations to the function of *PFN1* due to AD risk mutations could account for alterations in synaptic maintenance, leading to increased glutamate signaling to extra-synaptic NMDARs. *PFN1* activity is promoted by BDNF, which is hypothesized to be inhibited by extrasynaptic glutamate signaling, and loss of that signal could stop proper formation of actin at neurite outgrowths and potentially in astrocytic processes supporting synaptic clefts (Murk et al., 2012; Parsons and Raymond, 2014; Schweinhuber et al., 2015).

Another high-ranking hippocampal gene was *IQSEC1* (also known as *BRAG2*), which encodes a guanine nucleotide exchange factor, ARF-GEF_100_, that is critical for the proper maintenance of excitatory synapses through AMPA and NMDA receptor trafficking, and regulating synaptic long-term depression (Ottis et al., 2013; Elagabani et al., 2016; Um, 2017; Ansar et al., 2019) (Figure 6B and Table 1). Loss of function mutations in *IQSEC1* have been associated with intellectual disability (Elagabani et al., 2016) and a biallelic variant mutation has been observed in two families exhibiting intellectual disability and developmental delays (Ansar et al., 2019). A recent study in Wistar rats found that *BRAG2* is a member of a small network of proteins that are dysregulated in response to age-induced changes in proteostasis (Ottis et al., 2013). Significantly, changes in this protein network lead to impaired learning and memory performance (Ottis et al., 2013). Thus, common variants in *IQSEC1* could play a role in synaptic reorganization in response to aging and Aβ burden in AD.

The highest scoring amygdala gene was *PAK2*, which supports actin formation and the promotion of dendritic spine formation (Bokoch, 2003; Shin et al., 2009; Wang et al., 2018) (Figure 6C and Table 2). Mutations in *PAK2* are associated with other neurological disorders, including autism spectrum disorder and a 3q29 microdeletion syndrome with a range of neurological phenotypes including intellectual disability and autism (Wang et al., 2018). PAK family proteins have been associated with impaired dendritic spine formation in *in vitro* AD models (Ma et al., 2008), and PAK2 has been shown to be cleaved by caspase resulting in cell death (Marlin et al., 2011). Recent work has also shown that LIMK1, a downstream signaling molecule from *PAK2*, is involved in a ROCK2 actin regulatory pathway which mediates Aβ42-induced spine degeneration as well as neuronal hyperexcitability in hAPP mice (Henderson et al., 2019). *PAK2* activity is regulated by the Slit/roundabout (ROBO) signaling pathway (Dubrac et al., 2016; Xu et al., 2018), which is primarily involved in modulating axonal guidance and neuronal migration (Dickson and Gilestro, 2006; Mastick et al., 2010; Slováková et al., 2012), via the CDC42 GTPase (Wong et al., 2001; Xu et al., 2018; Huang et al., 2020). Another high-ranking amygdala gene, *SRGAP1*, suppresses the activity of *PAK2* through the Slit/ROBO signaling pathway (Dubrac et al., 2016; Xu et al., 2018) (Figure 6C and Table 2). Slit binds to ROBO and activates the SRGAP1 protein which triggers the hydrolysis of GTP by the CDC42 GTPase, which attenuates *PAK2* activity (Dubrac et al., 2016; Feng et al., 2016). Thus, common variants that modify the activity of *PAK2* or its upstream regulator, *SRGAP1*, could lead to alterations in synaptic morphology and axonal migration, and possibly to cleaved PAK2 signaling for neuronal death.

A final cytoskeletal protein among the top-rankings genes was *ENAH* in the amygdala. The ENAH protein has been found to form a complex with Fe65, a transcriptional activator and protein involved in neurite outgrowth and binding partner of amyloid precursor protein (APP) (Sabo et al., 2001; Li et al., 2018) (Figure 6D and Table 2). ENAH also binds to ROBO and profilin (PFN), acting as an inhibitor of motility and regulator of actin dynamics, respectively (Gertler et al., 1996; Lanier et al., 1999; Bear et al., 2000; Lanier and Gertler, 2000). Greater association of ENAH with the Fe65-APP complex supports neurite outgrowth and motility, whereas binding to ROBO inhibits that activity (Sabo et al., 2001). Common variants in *ENAH*, therefore, could influence synaptic plasticity through its association with the major AD risk factor *APP* (Trillaud-Doppia and Boehm, 2018).

### PRKCSH Potentially Regulates Excitotoxicity in AD

Loss of synaptic integrity coupled with impaired glutamate clearance by astrocytes caused by Aβ leads to high levels of extracellular glutamate, which binds to NMDARs increasing intracellular calcium levels (Parsons and Raymond, 2014; Liu et al., 2019). Under physiological conditions, the ER and other organelles act as calcium sinks that modulate intracellular ion levels.

Excitotoxicity occurs when intracellular calcium levels exceed the buffering capacity of the cell. The only top-ten gene shared by both tissues, aside from *APOE*, was *PRKCSH* (Tables 1, 2), which encodes the protein kinase C substrate 80K-H (80K-H), a glucosidase enzyme in the ER. 80K-H is known to colocalize with the inositol triphosphate receptor (IP3R), an ER-resident calcium channel that facilitates calcium currents in the ER (Kawaai et al., 2009) (Figure 6E). Common variants in *PRKCSH* could modify neuronal responses to excitotoxic levels of calcium, potentially exacerbating tissue atrophy in the hippocampus and amygdala.

### ER Stress and Misfolded Protein Response Genes Could Contribute to Apoptotic Signaling

Several other high-ranking genes are integral to the proper folding of proteins in the ER. ER stress occurs when the ability of the ER to properly fold proteins becomes saturated (Lin et al., 2007). The hippocampal gene *MOGS* encodes a glycosylation enzyme that aids in protein folding (Sadat et al., 2014; Li et al., 2019) (Figure 6F and Table 1). Common variants in *MOGS* could modify the rate at which ER stress occurs and exacerbate AD-related hippocampal atrophy.

When the ER reaches a critical state of misfolded proteins, ER-associated degradation (ERAD) can be triggered. ERAD is a process by which misfolded proteins are ubiquitinated and then proteolyzed to prevent the misfolded polymers from causing cellular damage. The amygdalar gene *UBE2J2* encodes a ubiquitin conjugating enzyme that marks misfolded proteins for degradation (Wang et al., 2009; Glaeser et al., 2018) (Figure 6F and Table 2). In some cases, ERAD can be triggered as part of apoptosis, and ubiquitination enzymes, including UBE2J2, are recruited to ubiquitinate misfolded proteins (Glaeser et al., 2018). Common variants in *UBE2J2* could affect the misfolded protein response and exacerbate cellular damage due to misfolded proteins.

### High Ranking Transcriptional Regulators Could Have Pleiotropic Effects on AD

A final set of high-ranking genes was broadly involved in transcriptional regulation. The high-ranking amygdala gene *EDF1* encodes a factor that acts as a transcriptional coactivator of peroxisome proliferator-activated receptor-gamma (PPARγ) (Figure 6G and Table 2). PPARγ has multiple functions, including regulating metabolism (Pipatpiboon et al., 2012), supporting vascular endothelial cells (Cazzaniga et al., 2018), and promoting BDNF expression (d’Angelo et al., 2019). It has been hypothesized that PPARγ counteracts insulin resistance and metabolic dysfunction in AD (Hoyer and Lannert, 1999; Pipatpiboon et al., 2012). It potentially also plays a role in modifying extracellular Aβ levels by facilitating increased uptake of Aβ by neurons and glia (Mandrekar-Colucci et al., 2012). PPARγ also downregulates the pro-inflammatory mechanisms of AD pathology (Combs et al., 2000; Govindarajulu et al., 2018). Common variants within the *EDF1* gene could have pleiotropic effects on cellular function through the regulation of PPARγ.

The hippocampal gene *HDAC3* encodes a histone deacetylase enzyme that epigenetically regulates gene expression (McQuown and Wood, 2011; Nott et al., 2016) (Figure 6H and Table 1). Extra-synaptic glutamate signaling drives pro-apoptotic gene expression, in part through the FOXO transcription factor, which is upregulated by extra-synaptic signaling (Parsons and Raymond, 2014). FOXO forms a complex with HDAC3, the protein product of *HDAC3*, and suppresses gene transcription (Nott et al., 2016). Thus, common variants in *HDAC3* could influence pro-apoptotic gene expression, exacerbating hippocampal atrophy.

HDAC3, and other members of the HDAC family, also negatively regulate long-term memory formation (McQuown and Wood, 2011; Zhu et al., 2017), via the “molecular brake pad hypothesis” (McQuown and Wood, 2011). The molecular brake pad hypothesis posits that the tight binding of HDACs to the promoters of genes that drive memory formation requires high-levels of activity-dependent signaling to dissociate them and enable protein synthesis-dependent long-term memory formation (McQuown and Wood, 2011). Notably, *HDAC3* has also been found to affect dendritic spine density, amyloid burden, microglial activation, and spatial memory in the *APP/PS1* AD mouse model (Zhu et al., 2017). Furthermore, in the 3xTG-AD mouse model, inhibition of HDAC3 reversed AD-related pathologies (Janczura et al., 2018), and in cultured rat hippocampal neurons, inhibition of HDAC3 reversed Aβ-induced plasticity deficits (Krishna et al., 2016). Interestingly, another histone deacetylase inhibitor, HDAC2, is emerging as a potential drug target in AD (Choubey and Jeyakanthan, 2018). Together, these results suggest pleiotropic roles for *HDAC3* as a gene influencing hippocampal atrophy in AD.

In summary, the genes prioritized by our integrative method are robustly related to AD by prior research and have clear pathways connecting them to neuron death, and therefore, to the imaging signals of low HV and AV.

The present study was potentially limited by a number of important factors. First, by treating HV and AV independently as quantitative traits, we potentially miss important population substructure (e.g., discrete patient subgroups with extreme neuropathology). While we do not see obvious subgroups in the HV/AV data (Figure 2), it is possible that by paring MRI with other phenotypic measures, such groups could appear. Future multi-trait analyses could have greater power to detect risk factors for patient subgroups, such as those that have been detected in gene expression data (Mukherjee et al., 2020). In particular, with emerging longitudinal data, it may become possible to identify subgroups that have distinct disease trajectories. Second, we have applied an NGR method that has been extensively tested, applied, and validated (Guan et al., 2010; Gorenshteyn et al., 2015; Goya et al., 2015; Greene et al., 2015; Krishnan et al., 2016; Song et al., 2016; Yao et al., 2018; Tyler et al., 2019). However, NGR methods are under active development, with new variants using different machine learning strategies or molecular networks. Future work can benchmark different NGR strategies prior to our integrative prioritization to identify the most robust combination of molecular network and learning algorithm for AD GWAS. Third, the present study focused on the genomic data alone. Neither the meta-GWAS or the ADNI-1 data in this study have gene expression for the study participants. However, gene expression data from patients with AD exist in other data sets, such as the Religious Orders Study (Bennett et al., 2012). Future work could integrate gene expression data into a prioritization pipeline, which has been done in other fields, such as cancer (Ritchie et al., 2013). Finally, we have not validated any of our gene candidates experimentally, and the proposed mechanisms for our highly ranked genes are speculative.

Despite the above limitations, however, the integrative approach we have taken has strongly implicated cytoskeletal dynamics, ER stress, and transcriptional dysregulation as major cellular processes driving neural atrophy. While it is beyond the scope of the present study to validate any of our candidates, by highlighting specific cellular processes and genes taking part in those processes, we can design robust *in vivo* and *in vitro* experiments to test them. For example, recent results in cultured neurons implicate impaired dendritic dynamics as a hallmark of AD (Froula et al., 2018; Boros et al., 2019; Henderson et al., 2019; Walker and Herskowitz, 2020; Walker et al., 2021). Such culture systems could be used for follow up experiments in which our candidate genes could feasibly be tested at scale.

## Data Availability Statement

Publicly available datasets were analyzed in this study. These data can be found here: http://adni.loni.usc.edu/data-samples/access-data/, https://ctg.cncr.nl/documents/p1651/AD_sumstats_Jansenetal_2019sept.txt.gz. We recognize that some may not be able to gain access to the ADNI data so we have made the gene-level summary statistics for the ADNI and MetaGWAS datasets available on the GitHub repository for this paper, along with all the code required to replicate this analysis: https://github.com/MahoneyLabGroup/AD_NBFP.

## Author Contributions

JB and JM designed the study. JB obtained the data and analyzed it using the pipeline designed by AT and JM. JB, ML, AT, and JM drafted and revised this manuscript. All the authors contributed to the article and approved the submitted version.

## Conflict of Interest

The authors declare that the research was conducted in the absence of any commercial or financial relationships that could be construed as a potential conflict of interest.

## Footnotes

1 adni.loni.usc.edu

2 https://www.cog-genomics.org/plink2/

3 http://faculty.washington.edu/browning/beagle/beagle.html

4 https://ctg.cncr.nl/software/magma

5 https://hb.flatironinstitute.org/download

6 https://gephi.org

## Supplementary Material

Supplementary Data Sheet 1 List of Hippocampus Volume Significant Genes.

Supplementary Data Sheet 2 List of Amygdala Volume Significatn Genes.

Supplementary Data Sheet 3 MetaGWAS Significant Genes.

Supplementary Data Sheet 4 Top Hippocampus Network Module GO Terms.

Supplementary Data Sheet 5 Top Amygdala Network Module GO Terms.

Supplementary Data Sheet 6 Final Hippocampus Scores.

Supplementary Data Sheet 7 Final Amygdala Scores.
